# Effect of Root Colonization by Arbuscular Mycorrhizal Fungi on Growth, Productivity and Blast Resistance in Rice

**DOI:** 10.1186/s12284-020-00402-7

**Published:** 2020-06-22

**Authors:** Sonia Campo, Héctor Martín-Cardoso, Marta Olivé, Eva Pla, Mar Catala-Forner, Maite Martínez-Eixarch, Blanca San Segundo

**Affiliations:** 1 Centre for Research in Agricultural Genomics (CRAG) CSIC-IRTA-UAB-UB, Campus Universitat Autònoma de Barcelona (UAB), Bellaterra (Cerdanyola del Vallés), Barcelona, Spain; 2 IRTA Institute of Agrifood Research and Technology, Field crops, Amposta, Spain; 3 IRTA Institute of Agrifood Research and Technology, Marine and Continental Waters, Sant Carles de la Ràpita, Spain; 4 Consejo Superior de Investigaciones Científicas, Barcelona, Spain

**Keywords:** Arbuscular mycorrhiza, *Funneliformis mosseae*, *Japonica*, *Magnaporthe oryzae*, Resistance, *Rhizophagus irregularis*, Rice, Symbiosis, Yield

## Abstract

**Background:**

Arbuscular mycorrhizal (AM) fungi form symbiotic associations with roots in most land plants. AM symbiosis provides benefits to host plants by improving nutrition and fitness. AM symbiosis has also been associated with increased resistance to pathogen infection in several plant species. In rice, the effects of AM symbiosis is less studied, probably because rice is mostly cultivated in wetland areas, and plants in such ecosystems have traditionally been considered as non-mycorrhizal. In this study, we investigated the effect of AM inoculation on performance of elite rice cultivars (*Oryza sativa*, *japonica* subspecies) under greenhouse and field conditions, focusing on growth, resistance to the rice blast fungus *Magnaporthe oryzae* and productivity.

**Results:**

The response to inoculation with either *Funneliformis mosseae* o*r Rhizophagus irregularis* was evaluated in a panel of 12 rice cultivars. Root colonization was confirmed in all rice varieties. Under controlled greenhouse conditions, *R. irregularis* showed higher levels of root colonization than *F. mosseae.* Compared to non-inoculated plants, the AM-inoculated plants had higher Pi content in leaves. Varietal differences were observed in the growth response of rice cultivars to inoculation with an AM fungus, which were also dependent on the identity of the fungus. Thus, positive, negligible, and negative responses to AM inoculation were observed among rice varieties. Inoculation with *F. mosseae* or *R. irregularis* also conferred protection to the rice blast fungus, but the level of mycorrhiza-induced blast resistance varied among host genotypes. Rice seedlings (Loto and Gines varieties) were pre-inoculated with *R. irregularis*, transplanted into flooded fields, and grown until maturity. A significant increase in grain yield was observed in mycorrhizal plants compared with non-mycorrhizal plants, which was related to an increase in the number of panicles.

**Conclusion:**

Results here presented support that rice plants benefit from the AM symbiosis while illustrating the potential of using AM fungi to improve productivity and blast resistance in cultivated rice. Differences observed in the mycorrhizal responsiveness among the different rice cultivars in terms of growth promotion and blast resistance indicate that evaluation of benefits received by the AM symbiosis needs to be carefully evaluated on a case-by-case basis for efficient exploitation of AM fungi in rice cultivation.

## Background

Arbuscular mycorrhizal fungi (AMF) are obligate biotrophs that establish mutualistic associations with roots of most terrestrial plants, including many crops (Smith and Read 2008; Parniske 2008; Bonfante and Genre 2010; MacLean et al. 2017; Choi et al. 2018). Root colonization by AM fungi improves the uptake of mineral nutrients in the host plant, mainly phosphorus and nitrogen, in exchange for photosynthetically fixed carbon. Along with this, root colonization by AM fungi has been shown to improve nutrition, ultimately helping in plant growth and development. Another benefit conferred by the AM symbiosis in several plant species is improved resistance to biotic and abiotic stress (Fritz et al. 2006; Pozo and Azcón-Aguilar 2007; Campos-Soriano et al. 2012; Nair et al. 2015; Cornejo et al. 2017; Wang et al. 2018; Rivero et al. 2018). Nevertheless, the benefits conferred by the AM symbiosis to the host plant vary depending on the identity of both the host plant (species and variety) and the AM fungal species (Sikes et al. 2009; Fernández et al. 2014; Taylor et al. 2015; Wang et al. 2016; Sawers et al. 2017; Watts-Williams et al. 2019). Furthermore, AM symbiosis is not always advantageous, as the effects can be positive, negative, or neutral to the host plant (Tawaraya 2003; Grace et al. 2009; Sawers et al. 2017; Watts-Williams et al. 2019). Environmental factors, such as soil properties, nutrient availability, or agricultural management practices, can also affect the association of crop plants with AM fungi. Hence, although AM fungi have been proposed as an alternative production practice to promote yield in crops, root colonization by AM fungi might not always be favorable for the host plant.

Legume species easily establish and benefits from AM symbiosis. Accordingly the legume species *Medicago truncatula* and *Lotus japonicus* have been widely used in studies on the AM symbiosis. Major cereal crops are also hosts for AM fungi, and the beneficial effects of the AM symbiosis are documented in maize, sorghum, oat, millet and wheat (Koide et al. 1988; Sawers et al. 2008, 2017, 2018; Beltrano and Ronco 2008; Ceasar et al. 2014; Watts-Williams et al. 2019).

Rice is one of the most important cereal crops in the world and a staple for more than half of the global population. Rice production systems include flooded and upland cultural systems, with a large predominance for the former because higher yields are obtained on flooded areas. However, rice production is severely threatened by the blast disease caused by the fungal pathogen *Magnaporthe oryzae* (Wilson and Talbot 2009). Traditional resistance (*R*) genes may confer blast resistance, but resistance conferred by *R* genes often breaks down in a few years due to the high variability and fast-evolving populations of this fungus. Moreover, it takes several years to introduce an *R* gene into a rice variety, even longer when pyramiding several *R* genes to generate new varieties with broader blast resistance. For these reasons, durable resistance to the rice blast fungus remains challenging, and the control of rice blast depends on the use of fungicides.

Evidence supports the natural colonization of rice by AM fungi in rice fields. Root colonization by AM fungi was reported in European rice varieties grown in aerobic conditions (Vallino et al. 2009). Here, all the checked rice varieties were colonized by AM fungi, the majority of AM fungal taxa being assigned to the *Rhizophagus irregularis* and *Funneliformis mosseae* (previously known as *Glomus intraradices* and *Glomus mosseae*, respectively) (Stockinger et al. 2009; Schüßler and Walker 2010). In other studies, rice plants grown in different locations in the southern United States exhibited natural colonization under non-flooded conditions (Bernaola et al. 2018a).

Although it is well known that rice can establish symbiotic associations with AM fungi, our current knowledge on the possible beneficial effects of the AM symbiosis in rice is still scarce. Most probably, this lack of knowledge is because the traditional rice farming systems involve growing rice in flooded (paddy) fields, and plants growing in aquatic environments were previously considered to be non-mycorrhizal. At present, however, this scenario has drastically changed, and the occurrence of AM fungi in roots of aquatic plants is well recognized (Lumini et al. 2011; Ruíz-Sánchez et al. 2011; Wang et al. 2011; Kohout et al. 2012; D’Souza 2016; Moora et al. 2016).

The AM fungus *R. irregularis* is one of the most widespread AM fungal species in the world. Evidence also support that *Rhizophagus irregularis* can grow and colonize rice plants in flooded soil while maintaining its functional capacities (Vallino et al. 2014). In other studies, the application of AMF at the nursery stage was found to increase yield by 14–21% in the wetland rice cultivar Nipponbare (Solaiman and Hirata 1997). In wetland rice Prakash, grain yield increased by 35–62% upon inoculation with *Acaulospora* sp., *Glomus fasciculatum*, or *G. mosseae* (Secilia and Bagyaraj 1994). On the other hand, we previously reported that root colonization by the AM fungus *R. irregularis* enhances resistance to the blast fungus *M. oryzae* in the rice cultivar Senia (*O. sativa*, ssp. *japonica*) (Campos-Soriano et al. 2012). Whether the AM symbiosis positively impacts blast resistance deserves further investigation. A better understanding of the impact of root colonization by AM fungi on rice yield and disease resistance will facilitate agricultural exploitation of the AM symbiosis in rice.

The aim of this research is two-fold. Firstly, to investigate the effect of inoculation with AM fungi (*R. irregularis*, *F. mosseae*) in a panel of temperate rice cultivars (*japonica* genotypes), focusing on plant growth and blast resistance. Secondly, to investigate whether the AM symbiosis can improve grain yield in rice cultivars grown under flooded conditions in experimental fields.

## Results

### Effect of AMF Inoculation on Growth of Rice Plants

**Fig. 1:**
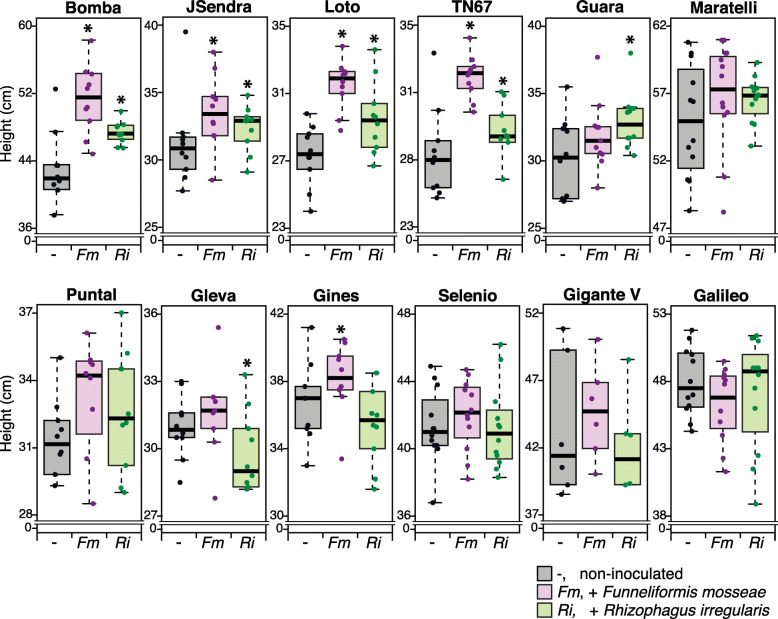
Growth of AM-inoculated rice varieties. Rice cultivars (temperate *japonica* genotypes) were inoculated with either *F. mosseae* or *R. irregularis*. Three weeks after inoculation with the AM fungus, plant height was measured (see Additional file 1: Figure S1a for the experimental design). Values in boxplots represent biological replicates (*n* = 12). Error bars indicate the first and third quartiles. The horizontal line within the box represents the median value (i.e., 50th percentile). Outliers are represented. Two independent experiments were carried out with similar results. Asterisks denote statistical differences (* *P* < 0.05, ANOVA test)

We conducted experiments to assess the phenotypic response to inoculation by an AM fungus, *Funneliformis mosseae* and *Rhizophagus irregularis*, in a panel of temperate *japonica* rice cultivars (see Additional file 1: Figure S1a for the experimental design). Depending on the rice variety, positive, neutral, or negative effects could be observed in response to AMF inoculation, which were evident as early as 3 weeks post-inoculation (wpi) with the AM fungus. Except for Galileo, inoculation with *F. mosseae* stimulated growth in all the rice cultivars assayed in this study (Fig. 1). Inoculation with *R. irregularis* also stimulated growth in seven out of the 12 varieties assayed, namely Bomba, JSendra, Loto, TN67, Guara, Maratelli, and Puntal (Fig. 1). Contrary to this, inoculation with *R. irregularis* had a negative effect on the growth of Gleva and Gines, whereas Selenio, Gigante Vercelli, and Galileo seemed to respond to inoculation by *R. irregularis* only slightly (Fig. 1). Growth stimulation in the AM-responsive varieties was more evident 10 weeks after inoculation (Additional file 1: Figure S1b).

Altogether, this study revealed a differential growth response to AM inoculation among rice cultivars, including positive, negligible, and even negative growth responses. The growth response to AM inoculation among rice varieties varied depending on the host genotype and the identity of the AM fungus, *F. mosseae* or *R. irregularis*.

### Accumulation of Inorganic Phosphate in Leaves of AM-Inoculated Rice Varieties

**Fig. 2:**
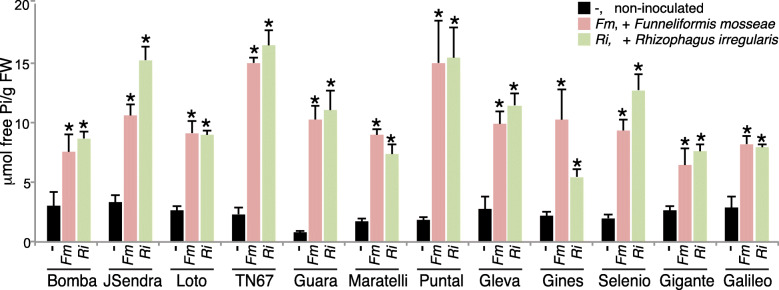
Pi content in leaves of AM-inoculated rice varieties. The panel of temperate *japonica* rice cultivars was inoculated with AM fungi (*F. mosseae* or *R. irregularis*). Pi content was determined from leaf fragments collected from plants four weeks after inoculation with AMF (see Additional file 1: Figure S1a for the experimental design). Data are mean ± SE (*n* = 5, each biological replicate is a pool of 2 individual leaves). Asterisks denote statistical differences (*p < 0.05, ANOVA test; AMF-inoculated vs non-inoculated). *Fm, Funneliformis mosseae; Ri, Rhizophagus irregularis*

Root colonization by AM fungi improves plant nutrition by increasing the availability and translocation of nutrients, specifically phosphate (Pi). To assess the functionality of the AM symbiosis, we analyzed the Pi content in leaves of mock-inoculated and AM-inoculated rice cultivars at 4 weeks after inoculation with either *F. mosseae* or *R. irregularis* (see Additional file 1: Figure S1a for the experimental design). Compared with the non-inoculated plants, inoculation with one or another AMF increased the Pi level in leaves of all the rice varieties here assayed (Fig. 2).

### Root Colonization by AM Fungi in Cultivated Rice Varieties

**Fig. 3:**
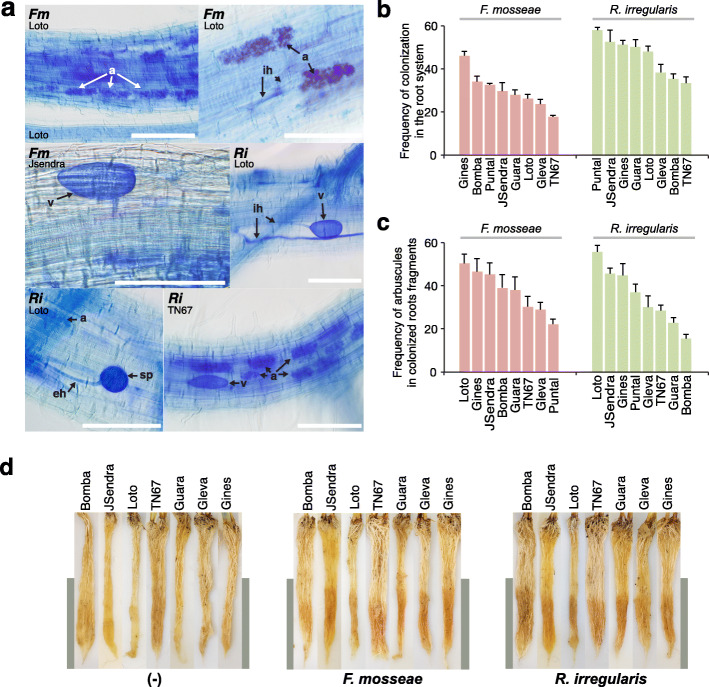
AM-colonization of temperate rice *japonica* rice cultivars. Roots from AM-inoculated temperate *japonica* rice cultivars were collected and analyzed (10 weeks post-inoculation). **a** Cotton blue staining of rice roots colonized by *F. mosseae* (*Fm*) or *R. irregularis* (*Ri*). The typical fungal structures were observed, such as extraradical hyphae (eh), intracellular hyphae (ih), arbuscules (a), vesicles (v), and spores (sp). Scale bar = 100 μm. **b-c** Estimation of AM fungal colonization according to the Trouvelot method **b** F%, frequency of mycorrhiza in the root system, and **c** a%, arbuscule abundance in mycorrhizal parts of root fragments. **d** Appearance of roots of rice plants inoculated with AM fungi. Representative pictures are presented. Increased pigmentation is observed in AMF-roots (grey bar)

To verify root colonization, we stained the roots of AM-inoculated plants with cotton blue. Microscopic observations revealed the presence of all the events related to fungal development in plants that had been inoculated with either *F. mosseae* or *R. irregularis.* They were: intraradical and extraradical hyphae, arbuscules at different morphological stages of formation, and vesicles (Fig. 3a). The overall colonization of rice roots inoculated with *F. mosseae* ranged from 46.1% (Gines) to 17.8% (TN67) (Fig. 3b), whereas *R. irregularis* colonization was higher, ranging from 58.1% (Puntal) to 33.4% (TN67) (Fig. 3b). However, the percentage of arbuscules in root fragments colonized by *F. mosseae* or *R. irregularis* was similar (Fig. 3c). Loto was the rice variety with the maximum arbuscule abundance (*F. mosseae*, 50.1%; *R. irregularis*, 55.4%) (Fig. 3c).

On the other hand, it is well known that the AM symbiosis activates carotenoid biosynthesis in several plant species (Strack and Fester 2006). Increased pigmentation in roots represents a visual marker for AM symbiosis establishment, including rice (Campos-Soriano et al. 2010). In most species, AM fungi colonize the elongation zone of roots (Mathesius 2003; Gutjahr et al. 2009). As expected, a higher yellow-orange pigmentation was observed in the elongation region of roots of plants that have been inoculated with either *F. mosseae* or *R. irregularis*, further supporting that the AM symbiosis is well established (Fig. 3d).

### Effects of Inoculation with an AM Fungus on Grain Yield in the Field

We investigated whether grain yield was affected in AM-inoculated rice varieties grown under field conditions. For this, the Loto and Gines cultivars were inoculated with the AM fungus *R. irregularis* and grown for 3 weeks under controlled greenhouse conditions to allow the establishment of the AM symbiosis. Then, AM-inoculated and non-inoculated plants were transplanted into freshly flooded paddy fields as described in Material and Methods (end of May, 2016), and allowed to grow for the whole season (see Additional file 2: Figure S2 for the experimental design). The panicles were harvested manually, and yield parameters were recorded.

**Fig. 4:**
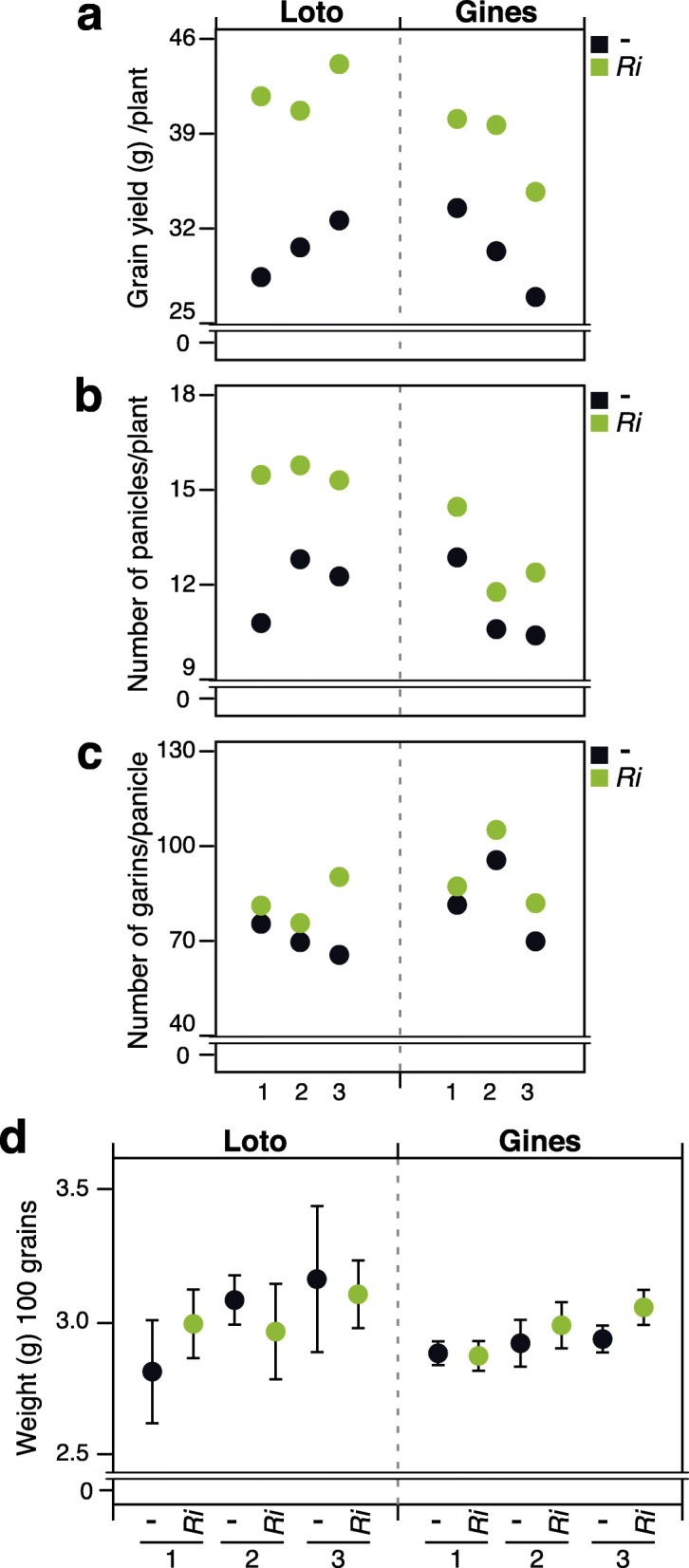
Effect of inoculation with *R. irregularis* on grain yield in field-grown rice plants. Rice plants (Loto and Gines) were grown in Ebro Delta (May–October 2016). For each variety, three independent plots (1 to 3) were designed for each condition (mycorrhizal: *Ri,* colored in green; non-mycorrhizal: -, colored in black) (see Additional file 2: Figure S2 for the experimental design). Grain yield parameters were measured. Twelve plants per condition in each parcel were analyzed and pooled. **a** Grain yield (grams) per plant. **b** Number of panicles in each plant. **c** Number of grains per panicle. **d** Grain weight. Measurements were done for 100 grains from plants from each variety and condition. Error bars represent the standard deviation for each condition

Interestingly, yields of Loto and Gines plants inoculated with *R. irregularis* were 41.61% and 28.68% greater than non-inoculated plants, respectively (Fig. 4a). Also, the number of panicles per plant increased by 30.13% and 14.77% in mycorrhizal Loto and Gines rice varieties, respectively (Fig. 4b). However, the number of grains in each panicle did not differ significantly between mycorrhizal and non-mycorrhizal plants (only a slight increase in the number of seeds/panicle could be observed in mycorrhizal plants) (Fig. 4c). When measuring grain weight, no statistical differences were observed between non-inoculated and *R. irregularis*-inoculated plants from each variety (Fig. 4d). These results demonstrated that inoculation with the AM fungus *R. irregularis* in the Loto and Gines rice varieties enhances grain production in the field, mainly by increasing the number of panicles.

These results also demonstrated that rice maintains functional AM symbiosis when grown under flooded conditions. In this respect, it has been reported that in rice roots, large lateral roots mostly support AM colonization, whereas fine lateral roots are not susceptible to colonization by AM fungi (Gutjahr et al. 2009, 2015; Vallino et al. 2014; Fiorilli et al. 2015). Although root colonization was confirmed in AM-inoculated rice plants grown under controlled conditions, in our hands, analysis of root colonization in field-grown rice plants was proven to be difficult using standard staining techniques (e.g. cotton-blue staining). It is also true that, compared with legume species, rice roots reach lower levels of root colonization by AM fungi, which together with a preferential colonization of large lateral roots in rice, might explain difficulties encountered for quantification of root colonization in field-grown rice plants.

### Resistance to Infection by the Rice Blast Fungus *M. oryzae* in Mycorrhizal Rice Plants

**Fig. 5:**
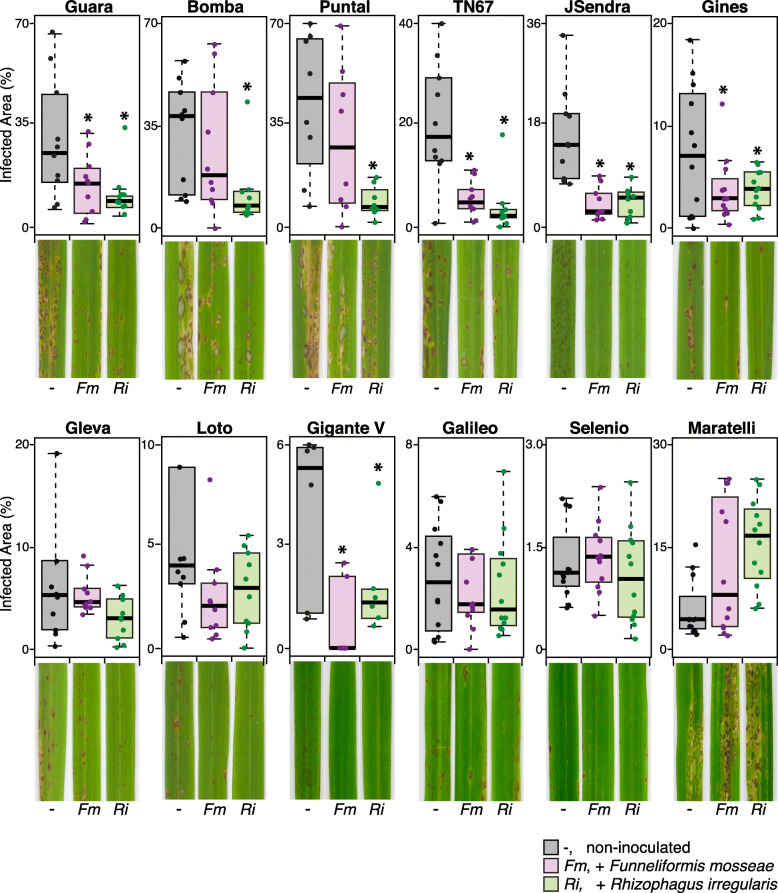
Resistance to *M. oryzae* infection of AM-inoculated rice. Rice seedlings (one-week-old) were inoculated with an AM fungus: *F. mosseae* or *R. irregularis*. Three weeks later, the rice seedlings were infected with *M. oryzae* spores (see Additional file 3: Figure S3 for the experimental design). Pictures show blast symptoms in each variety at the following times after *M. oryzae* infection: 4 dpi, Loto and Guara; 5 dpi, Puntal, TN67, Gleva and Maratelli; 6 dpi, JSendra, Gigante Vercelli, Gines, Galileo and Bomba, and 7dpi, Selenio. Three independent experiments were carried out with similar results. Box plots represent the percentage of leaf area affected by blast lesions as determined by image analysis. Values obtained for each biological replicate were plotted (*n* = 10). Error bars represent the first and third quartiles. The horizontal line within the box represents the median value (i.e., 50th percentile). Asterisks denote statistical differences (*p < 0.05, ANOVA test). Outliers are included. Representative images of *M. oryzae*-infected leaves from mycorrhizal and non-mycorrhizal plants are shown

In this study, we examined the resistance of mycorrhizal rice plants to infection by the foliar pathogen *M. oryzae*. For this, the different rice varieties in the panel were inoculated with either *F. mosseae* or *R. irregularis,* or non-inoculated and allowed to continue growth (controlled greenhouse conditions). At 3 weeks after AM inoculation, the rice seedlings were challenged with *M. oryzae* spores (see Additional file 3: Figure S3 for the experimental design). Disease symptoms were evaluated at different times after inoculation with *M. oryzae* spores. Visual inspection of mycorrhizal and non-mycorrhizal plants revealed a positive impact of AMF inoculation on blast resistance in most of the rice varieties here assayed, which was further confirmed by quantification of the leaf area showing blast lesions (Fig. 5). In particular, Guara, Bomba, Puntal, TN67, JSendra, and Gines that had been inoculated with either *F. mosseae* or *R. irregularis* developed less and smaller blast lesions compared to the corresponding non-mycorrhizal plants (Fig. 5, upper panel). A reduction in disease symptoms was also observed in AM-inoculated Gleva, Loto, and Galileo plants, but differences were not statistically significant.

In the case of Gigante Vercelli, this variety is known to show a basal level of blast resistance (Urso et al. 2016). In agreement with this, in our study, the non-mycorrhizal Gigante Vercelli plants developed small necrotic spots early during infection, but these necrotic spots did not progress with time. The mycorrhizal Gigante Vercelli plant, however, neither exhibited necrotic areas in response to *M. oryzae* infection nor developed the typical blast lesions (Fig. 5). We also noticed that the AM symbiosis increased blast susceptibility in Maratelli, a rice variety for which a high degree of susceptibility to *M. oryzae* infection has been described (e.g., in the absence of mycorrhizal root colonization) (Couch et al. 2005) (Fig. 5).

Overall, results obtained in this study revealed that the AM symbiosis confers protection to infection by the rice blast fungus, the magnitude of reduction of disease symptoms being dependent on the rice variety. However, the AM symbiosis might also have a negative effect on blast disease, as it is the case for the rice variety Maratelli.

## Discussion

In this study, we report the effect of AM fungal inoculation on growth responses and blast resistance in a panel of elite rice varieties (*japonica* subspecies). A commercial mycorrhizal inoculum of *F. mosseae* or *R. irregularis* was used in these studies. Growth responses and blast resistance were evaluated in plants grown under controlled greenhouse conditions. Importantly, the beneficial effects of AM inoculation of rice seedlings prevailed until harvest in rice plants growing in the field under flooding conditions. Here, pre-inoculation with *R. irregularis* results in an increase in grain yield in mycorrhizal Loto and Gines plants compared with non-inoculated plants.

Rice seedlings that had been inoculated with either *F. mosseae* or *R. irregularis* grew better compared to non-inoculated seedlings in most rice cultivars, a phenomenon generally known as positive Mycorrhizal Growth response (MGR) (Johnson and Graham 2013). It is generally assumed that the mycorrhiza-induced stimulation of plant growth is the consequence of improved nutrient uptake, generally phosphorus, as the AMF hyphal network allows access to a large soil surface area in the mycorrhizal root. This results in an increased phosphorus uptake through a mycorrhizal pathway of Pi acquisition (Smith and Smith 2011). However, our results demonstrated that there are rice varieties in which inoculation with one or another AM fungus results in growth retardation suggesting genotype-specific responses to AM fungi in rice. Growth inhibition in response to AM inoculation (negative MGR) has been previously reported in different plant species (Grace et al. 2009; Verbruggen et al. 2012). This negative effect is usually attributed to carbon losses from the host plant to the AM fungus with no subsequent gain in plant fitness from increased nutrient supply provided by the fungus (Grace et al. 2009; Johnson et al. 2015), particularly under high Pi conditions (Peng et al. 1993). Another hypothesis is that the suppression of the direct Pi uptake pathway in the host is not compensated by the mycorrhizal Pi uptake pathway (Smith and Smith 2011; Smith et al. 2011). Most probably, P acquisition in the various rice genotypes relies on the interplay between the direct and mycorrhizal P uptake pathways, the contribution of each pathway being dependent on the host genotype, the fungal species and Pi availability. However, our results indicated that Pi level increased in varieties positively responding to AM inoculation, as well in non-responsive or negatively responsive cultivars. On this basis, a differential growth response cannot be explained solely by a higher supply of Pi in mycorrhizal rice plants. In line with this, it has been reported that plant Pi concentration does not always increase with negative or positive MGR (Smith and Smith 2011). For example, significant differences in Pi uptake are found in *Medicago truncatula* colonized by three different AMF, all of them conferring positive MGR (Lendenmann et al. 2011). Also, negative MGR with increased Pi content is observed in *Glomus intraradices*-inoculated barley plants (Grace et al. 2009). The genetic basis of variations in AM-induced growth responses among host plant genotypes is still not well understood.

Although rice can have blast in all growth stages, blast incidence gradually decreases with the aging of plants as they develop adult plant resistance to leaf blast (Kim et al. 1987). Accordingly, in this work, the rice cultivars were screened for seedling blast resistance. Results obtained demonstrated that root colonization by *R. irregularis* or *F. mosseae* results in blast resistance to *M. oryzae* infection in most of the *japonica* rice cultivars assayed, namely Guara, Bomba, Puntal, TN67, JSendra, Gines, Gleva, Loto, Gigante Vercelli, and Galileo. Blast resistance in mycorrhizal plants occurs regardless of the level of root colonization in the various rice varieties. In other studies, different levels of *Glomus versiforme* colonization were observed in sorghum genotypes which conferred either beneficial or detrimental effects to the host plant, regardless of the level of root colonization (Grace et al. 2009). Several pieces of evidence support that root colonization by AM fungi confers disease resistance in several plant species, but most of these studies focused on resistance to root pathogens (Azcón-Aguilar and Barea 1997; Sikes et al. 2009; Jacott et al. 2017; Spagnoletti et al. 2020). Different results are found in the literature on the impact of the AM symbiosis on foliar pathogens which might well depend on the identity of the AM fungus, the host plant, and the interacting organism (Fritz et al. 2006; Pozo and Azcón-Aguilar 2007; Jung et al. 2012; Pozo et al. 2013; Jacott et al. 2017). Along with this, increased resistance, as well as increased susceptibility to leaf pathogens, were described in mycorrhizal plants (Liu et al. 2007; Pozo and Azcón-Aguilar 2007; Fiorilli et al. 2011; Song et al. 2015; Sanchez-Bel et al. 2016; Chen et al. 2019). In other studies, mycorrhizal rice plants were reported to be more susceptible to insect pests and infection by the sheath blight fungus *Rhizoctonia solani* (Bernaola et al. 2018b; Bernaola and Stout 2019). Also in rice, we previously reported that inoculation with *R. irregularis* in the rice cultivar Senia reduces blast disease symptoms (Campos-Soriano et al. 2012).

Recent studies demonstrated that AM colonization in roots of wild rice (*Oryza rufipogon*) was significantly higher than that in cultivated rice, the AM-inoculated wild rice being also more resistant to *M. oryzae* infection than AM-inoculated cultivated rice (Tian et al. 2019). It was proposed that the beneficial effects of the AM symbiosis might have been lost or reduced during rice domestication. Results here presented indicate that mycorrhizal-induced resistance (MIR) to infection occurs in elite *japonica* varieties, thus, confirming the potential of the AM symbiosis to improve blast resistance in cultivated rice. However, as there were also specific rice varieties in which AM inoculation increased blast susceptibility (e.g., Maratelli), the effectiveness on blast resistance in mycorrhizal rice plants must be evaluated on a case-by-case basis.

On the other hand, it is well known that rice plants maintain better growth and produce higher yields when grown in flooded conditions (paddy fields, anaerobic conditions), compared to plants grown in dry soil (aerobic conditions). Results obtained in this work demonstrated that pre-inoculation with *R. irregularis* results in a significant increase in grain yield in Loto and Gines plants that were grown in the field under flooded conditions. Here, it is worth mentioning that despite extensive research on the effect of AM inoculation in plant species growing in dry soil, the benefits received by AM symbiosis in plants under flooded conditions, in particular rice, remain less explored. Previously, it was reported that root colonization by AM fungi is reduced in rice plants grown under permanent flooded conditions (Lumini et al. 2011; Vallino et al. 2014). Evidence also support that the functional capacities of the AM symbiosis are not affected by flooding (Maiti et al. 2011; Vallino et al. 2014). Based on these findings, in this work, the rice seedlings were inoculated with the AM fungus and grown for 21 days under aerobic conditions prior to transplant into flooded fields. That the rice seedlings benefited from mycorrhizal colonization before transplanting was supported by the observed stimulation of plant growth and increased Pi content. Transplanted, AM-inoculated and non-inoculated rice plants, were then grown to maturity in paddy fields using conventional rice cultivation systems. Our results suggest that AMF inoculation at the seedling stage was beneficial for cultivated rice varieties after transplanting to flooded conditions in terms of productivity.

A unique characteristic of rice roots is the presence of large air spaces in mature roots, or aerenchyma, which provide an efficient air passage from shoots to roots (Colmer 2003; Rebouillat et al. 2009). As oxygen is provided by the aerenchyma in rice roots, the AM fungi that had entered into rice roots before flooding would remain viable in the roots in flooding conditions. Thus, although flooding reduces the initiation of colonization (Lumini et al. 2011; Vallino et al. 2014), once the fungus is established in the roots, it is able to maintain a functional relationship with the host plant in flooded conditions. This would explain why AM fungi are commonly present in rice roots (Secilia and Bagyaraj 1994; Solaiman and Hirata 1997; Zhang et al. 2016).

Although results here presented support that the mycorrhizal symbiosis increases rice yield in Loto and Gines varieties grown in paddy fields, this beneficial effect might depend on the stage of the fungus-root association when flooding occurred. From the perspective of practical application, the rational use of AM fungi in rice farming requires further investigation on how flooding might affect the establishment and functionality of the mycorrhizal association in rice. It will also be of interest to investigate whether seed inoculation with AM formulations, followed by direct seeding onto dry soil, is effective for the development of sustainable rice production systems while reducing water consumption.

In summary, our study demonstrated the potential of using AM fungi for increased yield in the elite rice cultivars Loto and Gines using conventional farming systems in paddy fields. Further studies would be needed to determine the extent to which the AM symbiosis improves production in other rice cultivars and whether inoculations with an AM fungus could also be one way to protect rice plants from the blast disease in the field. Modern rice farming largely depends on the input of fertilizers and pesticides to obtain maximum yields and to reduce losses due to pathogen infection. As a consequence, environmental problems have arisen in different rice-growing areas due to excessive and inappropriate use of fertilizers and pesticides. Results here presented open the possibility of using the AM symbiosis in rice cultivation, thus providing new opportunities to enhance rice yield and to promote sustainable agriculture.

## Conclusion

In this study, we report the influence of inoculation with arbuscular mycorrhizal fungi on growth and blast resistance of *japonica* rice cultivars. Twelve elite rice varieties were examined. All rice varieties were susceptible to root colonization by AM fungi: *F. mosseae* or *R. irregularis*. However, a substantial variation occurred in the plant growth response to AM inoculation among the different cultivars, this differential response being dependent on host genotype and identity of the fungus. Thus, positive, negative and neutral effects were observed on growth of mycorrhizal rice plants upon inoculation with either *F. mosseae* or *R. irregularis*. In most rice varieties, AM symbiosis conferred protection to infection by the rice blast fungus *Magnaporthe oryzae*. Mycorrhiza-induced resistance varied in the different rice genotypes. In the case of Maratelli, characterized as highly susceptible to blast, AM inoculation was found to enhance blast susceptibility. Field experiments revealed that pre-inoculation with *R. irregularis* substantially increased grain yield in Loto and Gines cultivars when grown under flooding conditions. Identifying the extent to which rice cultivars benefit from AM symbiosis in terms of growth, blast resistance, and productivity is crucial to exploit the AM symbiotic association in sustainable rice farming through reducing inputs that have environmentally negative impacts (i.e., fertilizers and pesticides). Due to the observed differential responses among rice cultivars, the evaluation of potential benefits received from the AM symbiosis needs be done on a case-by-case basis and in different environmental conditions.

## Materials and Methods

### Plant Material

Rice temperate *japonica* rice (*Oryza sativa*) cultivars were used in this study (named in the text as panel). They included cultivars grown in Spain (Guara, J. Sendra, Puntal, Gleva, and Bomba), Italy (Loto, Gigante Vercelli, Maratelli, Galileo, and Selenio) and France (Gines) (Courtois et al. 2012; Reig-Valiente et al. 2016). A Taiwanese cultivar Tainung 67 (TN67) closely related to Japanese and Korean temperate *japonica* varieties (Kim et al. 2018) was also included in this panel. Rice plants were grown in the greenhouse under controlled conditions (14 h/10 h day/night cycle, 28 °C/25 °C, and 60% humidity).

### Inoculation of AM Fungi and Growth of AMF-Inoculated Seedlings

Rice seeds were dehusked, surface-sterilized twice with 5% sodium hypochlorite for 15 min, and washed with sterile water minimum five times. Seeds were germinated on petri dishes with sterile water for 7 days. Then, germinating seedlings were transplanted to 150 ml-cones (20.5 cm; 2 plants/cone) containing a mix of 63.3% quartz sand (0.3–0.8 mm), 31.6% soil (turface and vermiculite 2:1), and 5% of either a granular inoculum of *Funneliformis mosseae* (formerly *Glomus mosseae*; FR140) or *Rhizophagus intraradices* (formerly *Glomus intraradices*; FR121), both commercially available (MycAgro; Bretenière, France; http://www.mycagrolab.com/). This granular inoculum is composed of mineral inert solid particles (clay, zeolite) and propagules of AM fungi (e.g., spores, mycelium and mycorrhizal root pieces) at a concentration of minimum 10 propagules/gram of granular inoculum. No inoculum was added to the substrate for the non-inoculated, control plants. A plastic cover was used to maintain high humidity during a 9-day acclimatization period. Transplanted seedlings were bottom-watered and allowed to continue growth under controlled conditions (Additional file 4: Figure S4). After this period, the plastic cover was removed and seedlings were top-fertilized with a modified Hoagland half-strength solution (2.5 mM KNO_3_, 2.5 mM Ca (NO_3_)_2_·4H_2_O, 1 mM MgSO_4_·7H_2_O, 0.5 mM NH_4_NO_3_, 25 μM KH_2_PO_4_, 23.15 μM H_3_BO_3_, 4.55 μM MnCl_2_·4H_2_O, 0.38 μM ZnSO_4_·7H_2_O, 0.1 μM CuSO_4_·5H_2_O, 0.14 μM Na_2_MoO_4_·2H_2_O, 26 μM Fe-EDDHA, pH 5.5) every 2 days (15 ml solution/cone) (Sánchez-Sanuy et al. 2019). Bottom-watering was continuously maintained. After 3 weeks of AMF inoculation, seedlings were grown according to the specific experiment (Additional file 4: Figure S4).

### Field Experiments

Field experiments were carried out to evaluate the effect of inoculation with *R. irregularis* on grain yield. Experiments were carried out at the IRTA (Institute of Agrifood Research and Technology) Experimental Station, Ebre Delta (Catalonia, NE Spain; May–October, 2016). Each experiment included two conditions: *R. irregularis-*inoculated and non-inoculated rice plants. Inoculation with *R. irregularis* and plant growth were carried out as described above (greenhouse, controlled conditions) (Additional file 2: Figure S2a). At 3 weeks after inoculation with the AM fungus, the rice seedlings were transplanted in irrigated rice fields (Additional file 2: Figure S2b). Three replications (plots) were performed, each one containing non-inoculated and *R. irregularis*-inoculated plants for each variety (Loto, Gines). These plots were located in different experimental fields in which commercial varieties were also grown (Additional file 2: Figure S2b). For each rice variety and condition (*R. irregularis*-inoculated, non-inoculated), 12 seedlings were transplanted in each of the three plots with a 3-m spacing. Thus, a total of 36 plants were grown for each variety and condition. Rice was grown in flooded conditions from 24 of May to 4 of October (2016). For evaluation of grain production, grains from the 12 plants of each individual plot, for each variety and condition were harvested and pooled.

### Analysis of Root Colonization

Root samples were collected at 10 weeks post-inoculation with AM fungi, extensively washed with sterile water, and analyzed for AM colonization with 0.1% cotton blue in acid lactic as previously described (Berruti et al. 2013). Roots were cut then into 1 cm fragments and mounted onto microscope slides. Estimation of mycorrhizal colonization was done from 75 root fragments (Trouvelot 1986). AMF structures were examined from cotton blue-stained roots an Axiophot Zeiss microscope equipped with a Digital color camera (DP70 Olympus) and 40X magnification.

### Blast Resistance Assays

The fungus *M. oryzae* (strain Guy-11) was grown on Complete Media Agar (CMA) supplemented with chloramphenicol (30 mg/L) in Petri dishes (9 cm) for 15 days at 28 °C. *M. oryzae* spores were prepared as described (Campo et al. 2013). Inoculation with AMF, and growth of non-inoculated or AMF-inoculated rice plants was done as described above (greenhouse, controlled conditions; Additional file 3: Figure S3). After 3 weeks, seedlings of either AMF-inoculated or non-inoculated were sprayed with a suspension of spores from the fungal pathogen *M. oryzae* (5 × 10^5^ spores/ml; 0.4 ml/plant) using an aerograph (pressure, 2 atm) (Sánchez-Sanuy et al. 2019). The inoculated seedlings were maintained in the dark overnight (at 90% humidity) and then allowed to continue growth under controlled conditions for the required time (Additional file 3: Figure S3). The percentage of leaf area affected by blast lesions was determined using the ImageJ/Fiji v2.00 software (http://fiji.sc/Fiji).

### Pi Content

Rice plants were AMF-inoculated or not, and grown under greenhouse conditions as described above (Additional file 1: Figure S1a for the experimental design). The youngest totally expanded leaves were harvested at 4 week after inoculation with one or another AM fungus. Five biological replicates were analyzed, each one consisting in a pool of two leaves obtained from two individual plants. Pi content of rice leaves was determined using a colorimetric as previously described (Ames 1966).

### Statistical Analyses

Means and standard errors were calculated using Microsoft Excel. Significant differences among non-mycorrhizal and mycorrhizal plants were assessed using ANOVA test (*p-value* ≤ 0·05).

## Supplementary information

**Additional file 1: Figure S1.** Experimental design used in this study for Growth and Pi analyses.

**Additional file 2: Figure S2.** Experimental design used in this study for Field Experiments.

**Additional file 3: Figure S3.** Experimental design used in this study for Blast resistance assays.

**Additional file 4: Figure S4.** Comparison of the different experimental designs used in this study.

## Data Availability

All data generated or analyzed during this study are included in this published article and its Additional files.

## Abbreviations

AM: Arbuscular mycorrhiza
AMF: Arbuscular mycorrhizal fungus
dpi: Days post-infection
wpi: Weeks post-inoculation
Pi: Inorganic phosphate
