# Supplementary material for: Effect of Root Colonization by Arbuscular Mycorrhizal Fungi on Growth, Productivity and Blast Resistance in Rice
Source: Rice (N Y). 2020 Jun 22;13:42. doi: 10.1186/s12284-020-00402-7 (PMC7310045; doi:10.1186/s12284-020-00402-7)
Supplement: Supplementary file 1 — Additional file 1: Figure S1. Experimental design used in this study for Growth and Pi analyses. [file 12284_2020_402_MOESM1_ESM.pdf]

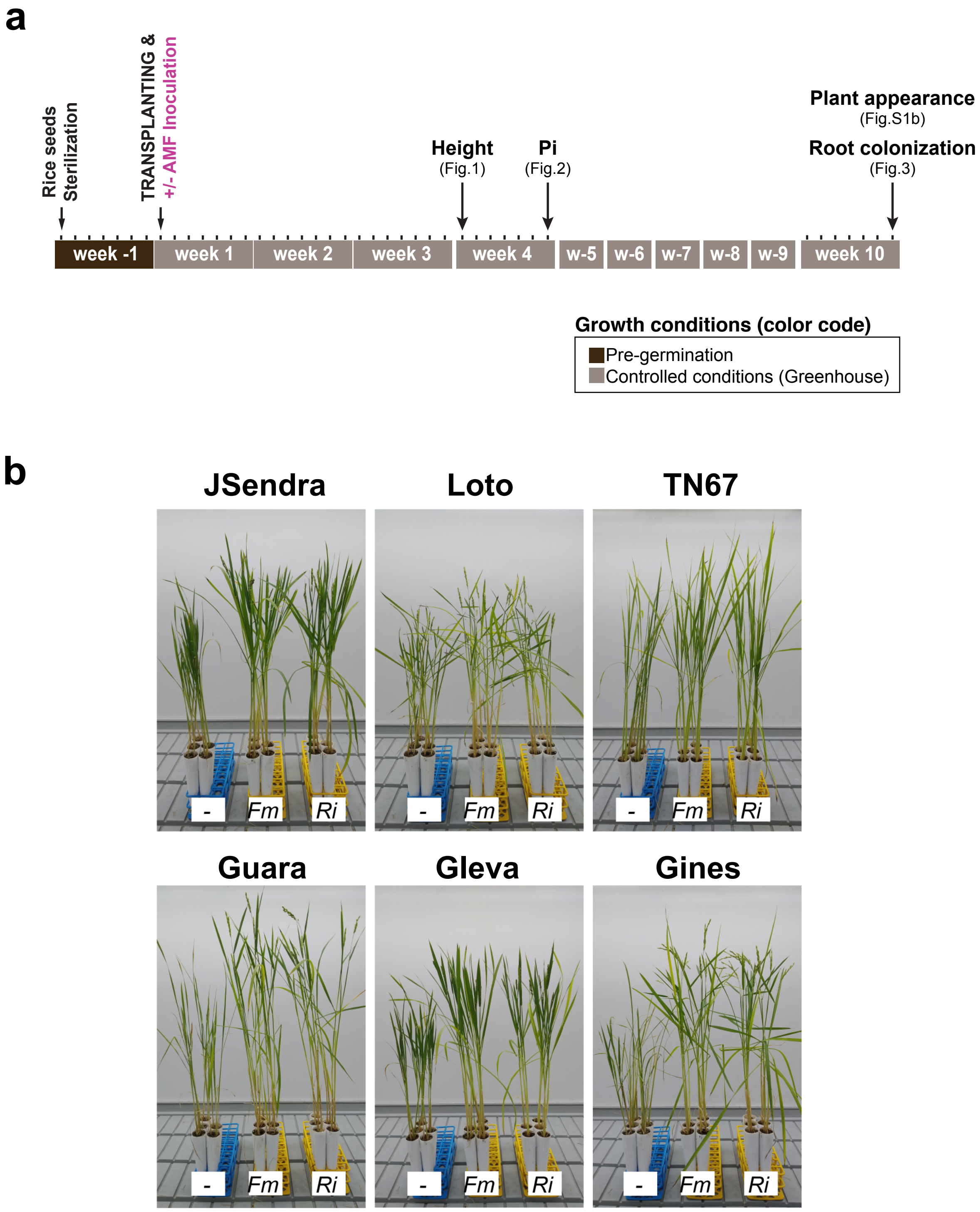

Additional file 1: Figure S1. Experimental design used in this study for Growth and Pi analyses
(a) Timeline for the experiments in AMF-inoculated and non-inoculated rice plants used in this study: Growth and Pi measurements. At later stages roots were collected and cotton-blue stained to verify AM colonization
(b) Appearance of AM-inoculated rice varieties. Plants were inoculated with the AM fungi *F. mosseae* (*Fm*) or *R. irregularis* (*Ri*), or mock-inoculated (-) and grown at the greenhouse under controlled conditions for 10 weeks (see Figure S1a). Representative images of the rice varieties responsive to AM inoculation are presented.
