# Supplementary material for: Effect of Root Colonization by Arbuscular Mycorrhizal Fungi on Growth, Productivity and Blast Resistance in Rice
Source: Rice (N Y). 2020 Jun 22;13:42. doi: 10.1186/s12284-020-00402-7 (PMC7310045; doi:10.1186/s12284-020-00402-7)
Supplement: Supplementary file 2 — Additional file 2: Figure S2. Experimental design used in this study for Field Experiments. [file 12284_2020_402_MOESM2_ESM.pdf]

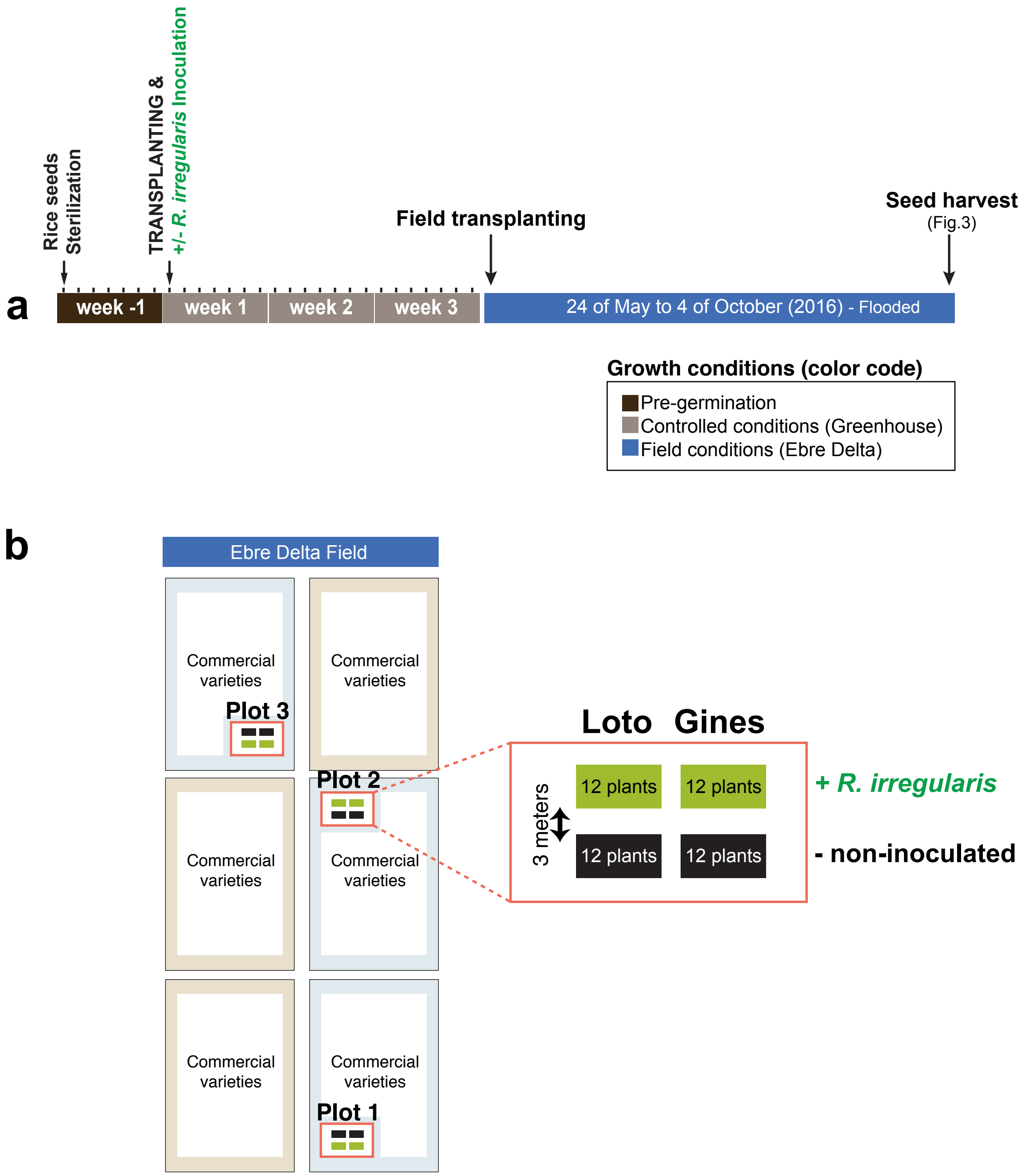

Additional file 2: Figure S2. Experimental design used in this study for Field Experiments
(a) Timeline for the experiment in AMF-inoculated and non-inoculated rice plants used in this study to analyze productivity in the field aunder natural conditions
(b) Experimental design in the field. Three plots (1-3, sorrounded with red line) were included for *Rizhophagus irregularis*-inoculated (colored in green) and non-inoculated (colored in black) Loto and Gines rice plants grown in paddy fields (blue-shaded squares). Commercial rice varieties were grown surrounding our experimental plots. An amplification of the experimental area for *R. irregularis*- and non-inocuated rice plants is shown. In all cases, non-inoculated and *R. irregularis*-inoculated plants were planted at a distance of 3 meters. 12 plants /condition/plot were analyzed (n=36 in total).
