# Supplementary figures and images for: Effect of Root Colonization by Arbuscular Mycorrhizal Fungi on Growth, Productivity and Blast Resistance in Rice
Source: Rice (N Y). 2020 Jun 22;13:42. doi: 10.1186/s12284-020-00402-7 (PMC7310045; doi:10.1186/s12284-020-00402-7)

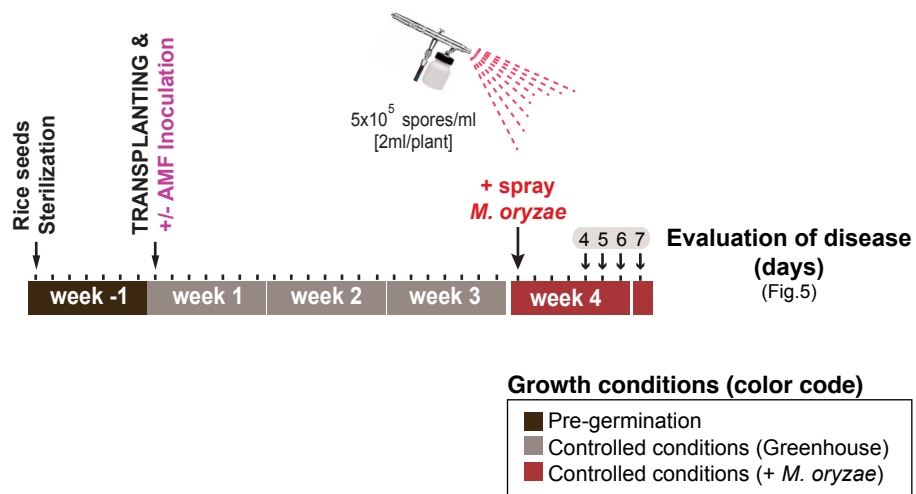

**Additional file 3: Figure S3. Experimental design used in this study for Blast resistance assays**

Supplement: Supplementary file 3 — Additional file 3: Figure S3. Experimental design used in this study for Blast resistance assays. [file 12284_2020_402_MOESM3_ESM.pdf]
