# Supplementary material for: Effect of Root Colonization by Arbuscular Mycorrhizal Fungi on Growth, Productivity and Blast Resistance in Rice
Source: Rice (N Y). 2020 Jun 22;13:42. doi: 10.1186/s12284-020-00402-7 (PMC7310045; doi:10.1186/s12284-020-00402-7)
Supplement: Supplementary file 4 — Additional file 4: Figure S4. Comparison of the different experimental designs used in this study. [file 12284_2020_402_MOESM4_ESM.pdf]

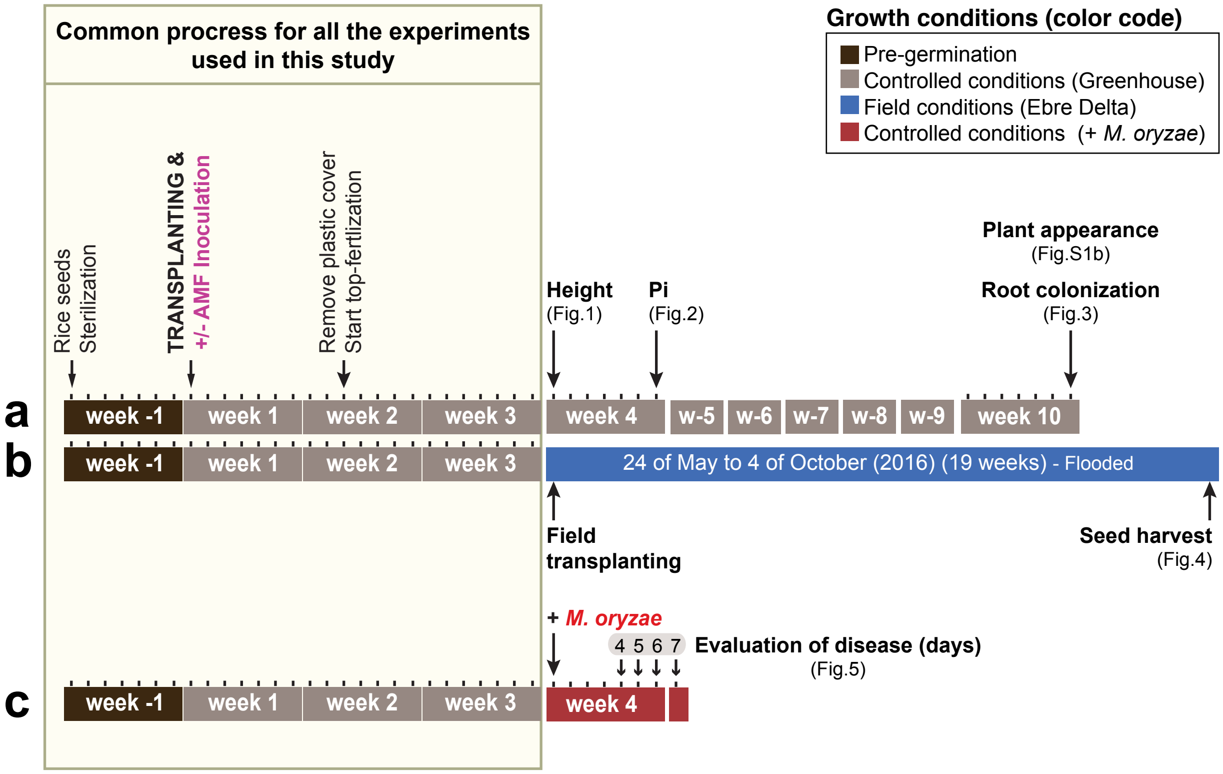

Additional file 4: Figure S4. Comparison of the different experimental designs used in this study (a-c) Timeline for the experiments in AMF-inoculated and non-inoculated rice plants used in this study (a) Growth and Pi measurements, (b) Productivity and (c) Blast resistance assays. The common protocol used in all the experiments used in this study is shaded in yellow, consisiting in the sterilization of rice seeds and pregermination in Petri dishes, and incoulation or not of rice seedlings with *F. mosseae* or *R. irregularis* during transplanting. A plastic cover was used to maintain high humidity during a 9-day acclimatization period. After this period, the plastic cover was removed and seedlings were top-fertilized with a modified Hoagland half-strength solution. Bottom-watering was continuously maintained from the intial transplanting.
